# Preparation, Characterization and *in vivo* Evaluation of Parenteral Sustained Release Microsphere Formulation of Zopiclone

**DOI:** 10.4103/0975-1483.66792

**Published:** 2010

**Authors:** N Swapna, AV Jithan

**Affiliations:** *Department of Pharmaceutics, Vaagdevi College of Pharmacy, Warangal -506 001, Andhra Pradesh, India*

**Keywords:** *In vitro* release, microspheres, pharmacodynamics, pharmacokinetics, polycaprolactone, zopiclone

## Abstract

The aim of this study was to prepare zopiclone-loaded polycaprolactone microspheres by emulsion solvent evaporation technique with different drug-to-carrier ratios {MP 1 (1:1), MP 2 (1:2), MP 3 (1:3), and MP 4 (1:4)}, characterize and evaluate the *in vivo* performance. The microspheres were characterized for particle size, surface morphology, drug excipient compatibility, percentage yield, drug entrapment, and *in vitro* release kinetics. Pharmacokinetics and pharmacodynamics were evaluated after parenteral administration so as to determine the sustained action of the drug after one-time administration of the formulation in a rat model. Of four formulations prepared, MP 2, i.e., 1:2 (drug–polymer) ratio was selected as the optimized formulation based on particle size, particle shape, and the release behavior. The size of microspheres was found to be ranging from 5.4 to 12.1 µm. The shape of microspheres was found to be spherical by SEM. Among the four formulations, MP 2 (1:2) showed maximum percentage yield of 75% ± 2.68%. There was no interaction between drug and polymer by FT-IR study. In the *in vitro* release study, formulation MP 2 (1:2) showed 86.5% drug release and was found to be sustained for 10 days. The microsphere formulations were able to sustain the release of drug both *in vitro* and *in vivo*. Pharmacodynamic study (Maze apparatus) indicated that the anxiolytic activity shown by zopiclone microspheres was significant when compared to the zopiclone solution given daily.

## INTRODUCTION

Zopiclone is a non-benzodiazepine, cyclopyrrolone derivative[1] with a chemical name[6-(5-chloropyridin-2-yl)-5-oxo-7*H*-pyrrolo[3,4-b]pyrazin-7-yl]4-methylpiperazine-1-carboxylate. Zopiclone exerts its action by binding on the benzodiazepine receptor complex[1] and modulation of the GABA_B_ Z receptor chloride channel macromolecular complex. As it is non-benzodiazepine,[2] it is nonaddicting, less sedating with no physical, or physiological dependence. Most commonly seen side effects are taste alteration or dysgeusia (bitter, metallic taste, which is usually fleeting in most users), dry mouth, and headache. The therapeutic and pharmacological properties of zopiclone include anxiolytic, anticonvulsant, hypnotic, and myorelaxant properties. For these purposes, it is administered daily via the oral route. Such an administration lacks patient compliance and is very often associated with side effects because of crests and troughs in the plasma levels. On the other hand, a parenteral sustained release dosage form like biodegradable microspheres encapsulating zopiclone can lead to effective therapeutic levels and reduced side effects for this drug and thereby can enhance its therapeutic utility. Thus, in this study we aimed to develop a sustained release microsphere formulation for zopiclone using polycaprolactone as the biodegradable polymer. After preparation of microsphere formulations using different drug–polymer ratios, the formulations were characterized for *in vitro* drug release, drug–polymer compatibility, size, and surface morphology.

The main objective of any drug therapy is to achieve a desired concentration of the drug in blood or tissue which is therapeutically effective and nontoxic for extended period of time, and this goal can be achieved by proper design of sustain release dosage regimen.[3,4] Thus, once we developed and characterized sustained release dosage forms using *in vitro* methods, we aimed to investigate its performance in an animal model. For this purpose, the developed microsphere formulations were administered via intraperitonial route, the pharmacokinetics and pharmacodynamics of the drug were investigated. The results are discussed.

## MATERIALS AND METHODS

### Materials

Polycaprolactone (average *M*_n_: 60000; average *M*_w_: 80,000; MP: 60 °C) was purchased from Sigma-Aldrich, Germany. Zopiclone was a gift sample from Natco Pharma, Hyderabad. Polyvinylalcohol was purchased from Qualigens Fine Chemicals (New Delhi, India). Dichloromethane was purchased from Finar reagents. To conduct *in vitro* drug release studies, magnetic stirrer and cyclo mixer from Remi Equipments Pvt. Limited were used. A SL 164 Elico Double Beam UV–Vis spectrophotometer was used to analyze the samples. For separation of plasma from blood, centrifuge (Remi industries) was used. HPLC of Cyberlabs was used to analyze the drug. Diethyl ether was obtained from Finar Chemicals, Ahmedabad. Maze apparatus was used to evaluate the anxiolytic activity. The protocol of all experiments was approved by the institutional animal ethical committee. Male Wistar rats (100–150 g, 5 to 6 weeks old) purchased from animal center of Mahaveera enterprises whose Registration No. is 146per1999perCPSCEA, Hyderabad were used in this study.

### Preparation of microspheres

Microspheres of zopiclone using biodegradable polycaprolactone as the polymer were prepared by emulsion–solvent evaporation method. Four different formulations MP 1, MP 2, MP 3, MP 4 containing drug–polymer in the ratio of 1:1, 1:2, 1:3, and 1:4, respectively, were prepared. Dichloromethane (10 mL) was taken as organic phase in which polymer and drug were dissolved.[2] The drug content was always 100 mg in all the batches. The aqueous phase contained polyvinylalcohol PVA solutions in water at 2% w/v (30 mL). The organic phase was added to the aqueous phase drop by drop while the aqueous phase was kept for stirring on a magnetic stirrer. Stirring was continued till complete evaporation of dichloromethane occurred. As the organic phase evaporates, precipitation of the polymer and drug occurs due to which drug gets entrapped in the polymer and stirring results in size reduction as well as spherical particle formation. The microspheres, thus obtained were filtered through Wattmann filter paper number I, and the free unentrapped drug was also removed. Microspheres were air dried and used for further studies.

### Physico-chemical evaluation of the microspheres

The microspheres prepared by emulsion solvent evaporation method[5] were evaluated for the following characteristics. The surface morphology and the internal textures of the microspheres were observed under scanning electron microscope. Particle size analysis was carried out using optical microscope ×40. About 50 particles were selected randomly, and their size was determined. Drug-excipients compatibility study[6] was carried out by the FTIR analysis of pure drug (zopiclone), pure polymer (polycaprolactone), microparticular system (zopiclone MS), and placebo microparticles (polycaprolactone MS). High-performance liquid chromatographic analysis was used for estimation of drug content.

#### *In vitro* release studies

The dialysis bag diffusion technique is widely used to evaluate drug release from micro- and nanosized carriers.[7] This technique with the help of a dialysis membrane was used in this study. This membrane was then carefully clamped to one end of the hollow glass tube and considered as the donor compartment. The dissolution medium phosphate buffer pH (7.4)—50 mL was taken into the receiver compartment. The donor compartment was immersed into the receiver compartment so that the edge just touches the receiver compartment. An aliquot of 100 mg of the microparticles were dispersed in 1% NaCMC in phosphate buffered saline (PBS) solution and placed in the donor compartment. The rpm of the system was maintained at 50 by using magnetic stirrer and bead. Samples (5 mL) were removed from the receptor compartment and replaced with fresh medium immediately. These were analyzed by spectrophotometric method at 303 nm wavelength.

#### Kinetics of drug release[3]

In order to understand the mechanism and kinetics of drug release, the result of the *in vitro* dissolution study of microspheres were fitted with various kinetic equations, such as zero-order (percentage release versus time), first-order (log percentage of cumulative drug remaining versus time), Higuchi’s model (percentage drug release versus square root of time), and Korse Meyer Peppas model (log cumulative amount versus log time). Correlation coefficient (*r*^2^) values were calculated for the linear curves obtained by regression analysis of the above plots. Further, to confirm the mechanism of drug release, the first 60% of drug release was fitted in Korsemeyer–Peppas model with the equation: *M*_t_ /*M*_∞_. = *Kt*, where *M*_t_ /*M*_∞_ is the fraction of the drug release at time t, the n value is used to characterize different release mechanisms and is calculated for the slope of the plot of the log of fraction of drug released versus log of time (*t*).

### Evaluation of pharmacokinetics of zopiclone in rats

The objective of this study was to determine the plasma concentration profile of zopiclone after administrations of its solution form after intravenous administration, suspension form after oral administration as well as in the microsphere form after intraperitoneal administration and evaluate the benefits of the microsphere formulation in terms of plasma concentration profile. In this study, the i.v. solution administration as well as i.v. oral administration are the references. The doses administered in the intravenous as well as oral administrations are equivalent doses to that administered in the form of microspheres. Microspheres containing 15 mg of zopiclone for prolonging the drug release in the plasma for 10 days with equivalent oral and i.v. dose of 15 mg were administered. The study was carried out on male Wistar rats weighing 100–150 g. After procurement of rats, these were acclimatized for 10 days prior to the conduction of the experiment. The animals were housed in clean cages and maintained in controlled temperature. They were fed with standard diet and *ad libitum*. Animals were divided into four groups. Each group contained four rats.

**Table:** 

*Group 1:*	Received normal saline solution (0.9 mg/mL of NaCl) orally every day (1 mL/rat).
*Group 2:*	Received 15 mg of zopiclone suspension (1% NaCMC) orally.
*Group 3:*	Received 15 mg of zopiclone solution by intravenous route.
*Group 4:*	Received zopiclone microspheres equivalent to 15 mg of drug suspended in normal saline (0.9% w/v) solution intraperitonially on day 1.

Blood samples (0.5 mL) were collected from retro orbital sinus of rat eye under anesthesia at intervals of 1, 3, 6, 12, and 24 h. For microparticular system, samples were collected after 24 h (i.e., day 1) day 2, day 3 till 10 days, respectively. The blood samples so collected were added to a series of graduated microcentrifuge tubes containing 0.3 mL of sodium citrate solution (4% w/v in water). All the samples were centrifuged at 3000g for 10 min, and plasma was separated into other microcentrifuge tube by using micropipette and stored in deep freeze. The drug was extracted from the plasma by adding 500 µL of diethyl ether,[8] and vortexed on cyclo mixer for 20 min. The organic phase was separated and collected into another microcentrifuge tube and allowed to air dry by keeping the lid of the tube open for 24 h. These dried tubes were stored in deep freeze until HPLC analysis was performed. Before the HPLC analysis, samples were reconstituted with 50 µL of mobile phase (methanol–water, 40:60) and analyzed at 304 nm wavelength. Prior to this, a HPLC standard curve for the drug in the plasma was generated. The mobile phase consisted of methanol–water at a ratio of 40:60.

### Evaluation of pharmacodynamic (anxiolytic) activity of zopiclone

This study was designed to determine pharmacodynamic effects with the one time microsphere formulation and compare with daily administration of a similar dose. As a reason, the dose contained in the microspheres is for the entire 10 days (1.5 × 10 days =15 mg). The study was conducted on 16 Wistar rats, which were divided into four groups each group containing four rats.

**Table:** 

*Group 1:*	Served as normal control and received normal saline solution intraperitonially daily (1 mL/rat).
*Group 2:*	Served as standard and was given 0.5 mg/kg of diazepam intraperitonially every day. The compound was dissolved in 0.9% saline by solubilization in one or two drops of 1 N HCl. Drug solution pH was then adjusted to approximately seven with 1 N NaOH.
*Group 3:*	Received zopiclone solution at a dose of 1.5 mg daily for 10 days intraperitonially. The drug was dissolved in 0.9% saline by solubilization in one or two drops of 1 N HCl. Drug solution pH was then adjusted to approximately seven with 1 N NaOH.
*Group 4:*	Received zopiclone microspheres equivalent to 15 mg of drug suspended in normal saline (0.9%w/v) intraperitonially on day 1.

All the preparations, vehicle, or diazepam were administered by intraperitonial route 45 min before start of session. To start session rat was placed at the center of maze, its head facing closed arm and allowed to explore for 5 min.[9,10] During this, 5 min time spent in open arm and number of entries in open arm were recorded. An entry was defined as all four paws on the arm.

## RESULTS AND DISCUSSIONS

**Figure 1:**
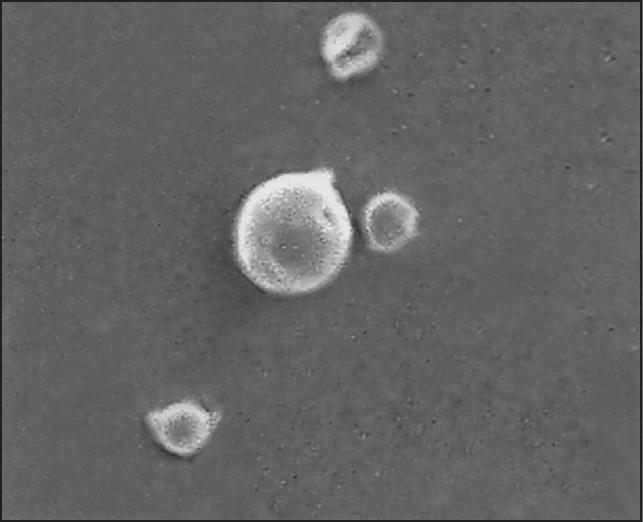
SEM picture of zopiclone microspheres (magnification, ×2000)

**Figure 2:**
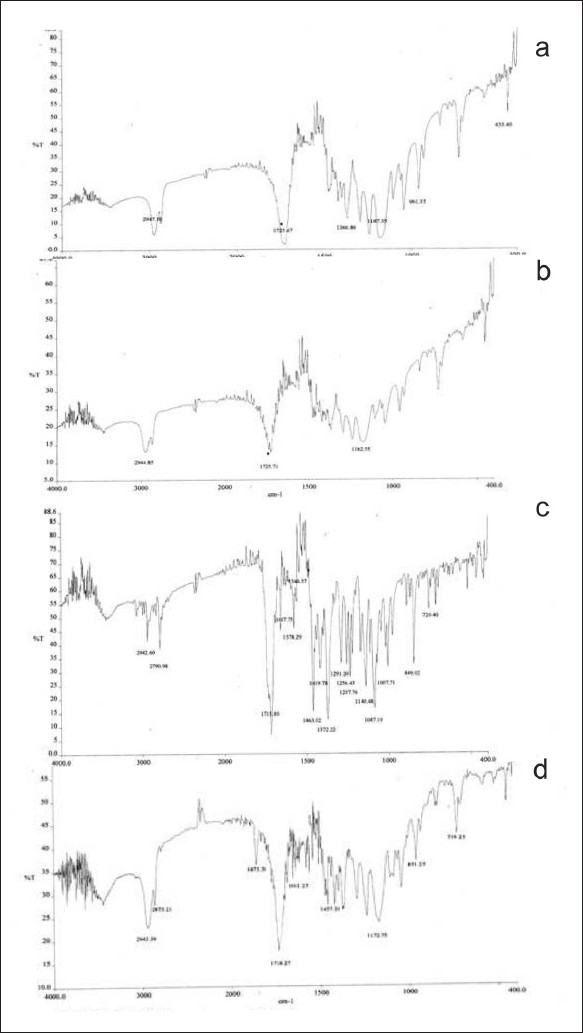
FTIR spectra (a) zopiclone; (b) polycaprolactone; (c) placebo microspheres; and (d) zopiclone microspherespreparation

Polycaprolactone microspheres[11] of zopiclone were prepared by emulsion solvent evaporation technique. Polycaprolactone was selected as a polymer for the preparation of microspheres because of its biodegradable and biocompatible properties. Four different formulations MP 1, MP 2, MP 3, and MP 4 were prepared. Out of these formulations MP 2, i.e., 1:2 (drug–polymer) ratio was selected as the optimized formulation based on particle size, particle shape, and the release behavior. The microspheres were found to be spherical with quite smooth surfaces when viewed microscopically. Scanning electron microscopy of the microspheres showed that microspheres have spherical shape and discrete (PCL) [Figure 1]. The percentage yield was high for all the formulations and were in the range of MP 1 (50%), MP 2 (75%), MP 3 (72%), and MP 4 (74.5%), respectively. The particle sizes were differed due to variation in the composition of the formulation. Zopiclone was found to be compatible with the polymer in the microparticular formulation. This compatibility was concluded by comparing the FTIR profiles of pure polymer, pure drug, placebo biodegradable microparticles, and zopiclone biodegradable polycaprolactone microspheres. Spectra were shown in Figure 2. From the spectra, it was clear that there is no interaction between the drug zopiclone and polymer polycaprolactone at the end of fabrication of microspheres. Entrapment efficiency of the drug in MP1, MP2, MP3, and MP4 were found to be 32% ± 33%, 40% ± 12.3%, 35% ± 5.5% and 28% ± 1.11%, respectively. The preparation method in this study was selected based on its suitability for poorly soluble drugs, and it was anticipated that the encapsulation would be appropriate using this methodology. Zopiclone is a poorly soluble drug. However, the encapsulation efficiencies were lower. This suggests that either the surfactant was not appropriate or enough drug was not partitioned into the polymer during the fabrication process. The state of art methodologies in such cases is to identify newer or appropriate surfactants and also to use polymers in which the drug is highly or appropriately encapsulated. If required newer methods of encapsulation such as o/o method, etc., might be of help. The objective of this study was to determine the feasibility of preparation of biodegradable parenteral microsphere formulations for zopiclone using solvent evaporation technique and perform the *in vivo* evaluation with the developed microspheres. In our subsequent studies, we would aim to improve the formulation aspects of zopiclone microspheres.

**Figure 3:**
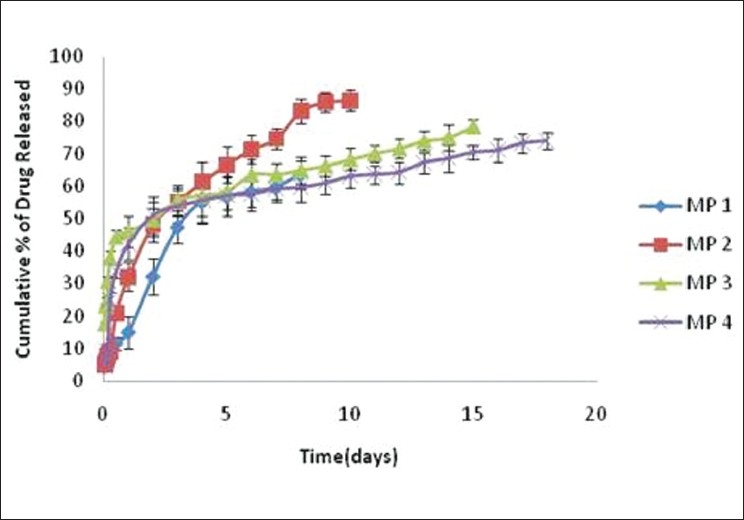
*In vitro* drug release of zopiclone from microsphere formulations.

*In vitro* drug release from the zopiclone microspheres was carried out by dialysis membrane technique. The cumulative percent release of zopiclone from various formulations was shown in Figure 3. The cumulative drug release of various formulations was found to be MP 1 (63.5%), MP 2 (86.4%), MP 3 (78.4%), and MP 4 (73.9%). The drug release from MP 1 was sustained for only 8 days and may be due to insufficiency of polymer to drug. In case of MP 4, the drug release sustained for 18 days but cumulative percentage release was found to be less compared to MP 2 due to high polymer content unabling the drug release probably because of drug binding to the polymer.

The drug–polymer ratio 1:2 showed better release pattern. It controlled the drug release for 10 days and was found to be the most suitable among other formulations.

The *in vitro* release data were applied to various kinetics models to predict the drug release mechanism and kinetics [Table 1]. The best-fit with the highest correlation coefficient was shown in Higuchi, first-order and followed by zero-order equations as given. The drug release mechanism from the microspheres thus can be described as diffusion controlled. The drug release was proportional to square root of time. When percentage of cumulative drug released versus time was plotted in accordance with first-order and zero-order equations, the *r*^2^ values obtained were found to be better for first-order plot compared to zero-order drug release, indicating that the drug release was described better with first-order release kinetics. The data obtained were also put in Korsmeyer–Peppas model in order to find out *n* value, which describes the drug release mechanisms. The *n* value of microspheres of different drug-to-polymer ratio was ranged between 0.23 and 0.54, indicating that the mechanism of drug release was diffusion controlled. The release also showed high correlation with the Korsmeyer–Peppas model.

**Table 1:** Value of *r*^2^ from release data of various formulations for different models of drug release

Model	MP 1	MP 2	MP 3	MP 4
Zero-order	0.92	0.91	0.90	0.84
First-order	0.99	0.97	0.98	0.94
Higuchi Korse Meyer	0.969	0.987	0.933	0.869
Peppas	0.973 (*n* = 0.33)	0.982 (*n* = 0.25)	0.960 (*n* = 0.45)	0.894 (*n* = 0.53)

The values listed are the values of coefficient correlation (*r*^2^) obtained from release data of various formulations for different models of drug release, and *n* is the release exponent of Korsmeyer–Peppas model

**Figure 4:**
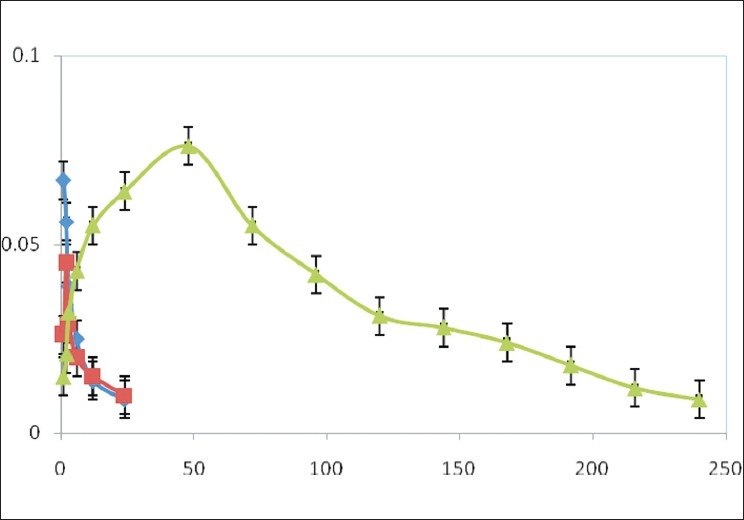
Plasma concentration time profile of zopiclone after oral, IV and IP administrations [Key: ■oral suspension administration; ♦intravenous solution administration; ▲intraperitoneal microsphere administration)

Drug levels in the plasma were determined using HPLC. The retention time of the drug was 9.2 min. The UV–Vis detection wavelength was 304 nm. Plasma drug concentration versus time profile given in different routes were shown in Figure 4. Microspheres sustained the release of the drug up to 10 days, reflecting in an increased area under curve.

**Figure 5:**
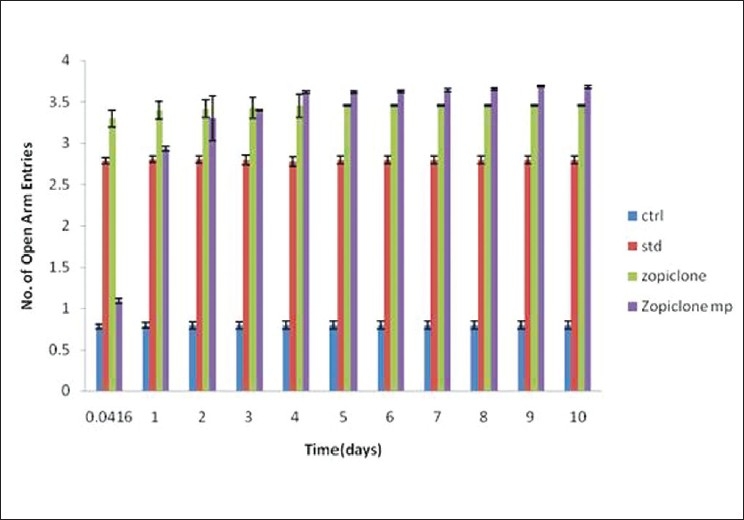
The effects of diazepam, zopiclone, zopiclone microspheres on rats (open arm entries in rectangular maze)

**Figure 6:**
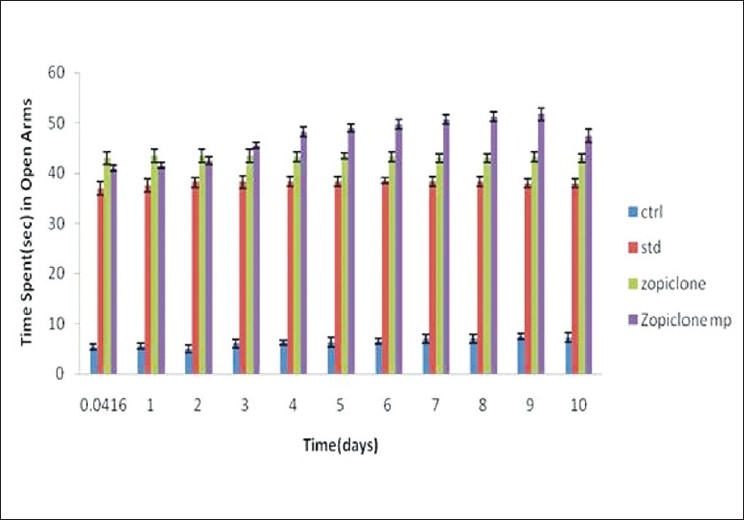
The effects of diazepam, zopiclone, zopiclone microspheres on rats (time spent in open arm in rectangular)

Anxiolytic activity[9,10] of zopiclone microspheres was evaluated using maze apparatus in which number of entries in open arm and time spent (s) in open arm were considered. The number of entries in open arm and time spent (s) in open arm were noted for standard diazepam administered daily through intraperitonial route, pure drug, i.e., zopiclone solution administered daily intraperitonially (10 mg/kg) and for zopiclone microspheres administered once starting day of the experiment [Figures 5 and 6]. All these results clearly indicate that the number of entries in open arm and time spent in open arm for zopiclone microspheres were significant when compared to the zopiclone solution given daily.

## CONCLUSION

In this study, an attempt made to prepare zopiclone microspheres using a biodegradable, biocompatible carrier, polycaprolactone by emulsion solvent evaporation technique. The method was found to be simple and reproducible. These systems provide better therapeutic efficacy owing to continuous availability of drug in therapeutic ranges over a long period of time. Microspheres sustained the drug release both *in vitro* and *in vivo*. Data conveniently suggest that the microsphere formulation is more effective than the drug in conventional solution form. It may be concluded from the result obtained from evaluation that these systems are useful in achieving controlled drug release profile and help to reduce dose of the drug, dosing frequency, unwanted toxicities, and improved patient comfort and patient compliance. Thus, in this study a sustained release parenteral formulation of zopiclone has been developed and this formulation was evaluation both *in vitro* and *in vivo* for sustained release of the drug.
